# Impact of a ketogenic diet intervention during radiotherapy on body composition: I. Initial clinical experience with six prospectively studied patients

**DOI:** 10.1186/s13104-016-1959-9

**Published:** 2016-03-05

**Authors:** Rainer J. Klement, Reinhart A. Sweeney

**Affiliations:** Department of Radiation Oncology, Leopoldina Hospital Schweinfurt, Robert-Koch-Straße 10, 97422 Schweinfurt, Germany

**Keywords:** Cancer, Ketogenic diet, Radiotherapy, Bioimpedance analysis

## Abstract

**Background:**

Based on promising preclinical data, ketogenic diets (KDs) have been proposed as supplementary measures for cancer patients undergoing standard-of-care therapy. However, data is still scarce on the tolerability and effects of KDs on cancer patients undergoing radiotherapy (RT). Here we present six cases of patients who underwent RT and concurrently consumed a self-administered KD in our clinic within a busy community hospital setting.

**Methods:**

All patients were followed prospectively with measurements of blood parameters, quality of life and body weight and composition using bioelectrical impedance analysis.

**Results:**

No adverse diet-related side effects occurred. Two patients had no elevated ketone body levels in serum despite self-reporting compliance to the diet. There was consensus that the KD was satiating and weight loss occurred in all patients, although this was only significant in two patients. Our data indicate that weight loss was mainly due to fat mass loss with concurrent preservation of muscle mass. Overall quality of life remained fairly stable, and all subjects reported feeling good on the diet. Tumor regression occurred as expected in five patients with early stage disease; however one subject with metastatic small cell lung cancer experienced slight progression during three cycles of combined chemotherapy + KD and progressed rapidly after ending the KD.

**Conclusions:**

Our data lend support to the hypothesis that KDs administered as supportive measures during standard therapy are safe and might be helpful in preservation of muscle mass. Further studies with control groups are needed to confirm these findings and address questions regarding any putative anti-tumor effects. Based on the experience with these six cases we implemented further steps to improve issues with KD compliance and initiated a clinical study that is described in a companion paper.

## Background

The term “ketogenic diet” (KD) generally describes any diet under which the serum levels of the ketone bodies β-hydroxybutyrate (BHB), acetoacetate (AcAc) and acetone increase beyond their reference ranges, typically 0.3 mmol/l for BHB, the most abundant circulating ketone body. This state of “ketosis” is a physiological condition for humans also occurring naturally in neonates, during intermittent and longer-term fasting or after intense exercise without glucose replacement [1]. To achieve the state of “nutritional ketosis”, a KD is usually composed of a very low carbohydrate (CHO) and high fat content and may additionally be combined with calorie restriction.

In recent years, the number of expert reviews concluding that KDs possess promising effects for cancer patients has sharply increased [2–10]. Specifically, it has been proposed that ketone bodies would confer protection to normal cells during radio- or chemotherapy while being non-protective or even toxic to tumor cells [6]. In addition, a high fat diet with ample protein intake may be ideally suited to meet the metabolic demands of the cancer patient whose metabolism is often affected by the tumor in such a way that peripheral tissues become more CHO “intolerant” while relying more on fatty acid oxidation [10]. However, clinical research on the effects of KDs in cancer patients is still scarce. There are some concerns against using KDs in cancer patients due to the side-effects that can occur. These include deficiencies in micronutrients, appetite loss, nausea, constipation, fatigue, hyperlipidemia and—especially relevant for cancer patients weight loss [11]. Some of these side-effects, however, may be attributable to the transition phase in which the body has to adapt to the high fat and low CHO content of the diet, usually one to 3 weeks. Other, longer-term side-effects may be preventable by restricting the KD to the timeframe of (RT)/radiochemotherapy (RCT) since there is evidence that it is most useful as a supportive intervention in this context [6]. We have therefore started to collect data on ambulatory patients consuming a KD during RT, and here report our initial clinical experience with the first six of such patients within a busy community hospital environment. Our main aim was to assess the feasibility of such an intervention and track body composition changes during the combined KD-RT treatment; secondary outcome variables included biochemical blood parameters and quality of life (QoL). While these data are unable to provide information about putative anti-tumoral effects of the diet, they might add further evidence to the hypothesis that KDs in cancer patients are safe and feasible as supportive measures during RT.

## Methods

Prior to the initiation of this study we received approval by the institutional ethics review board. Data collection was performed on the first six consecutive patients undertaking a KD during RT/RCT in our clinic. In most cases patients already had initiated or wished to initiate the diet at the initial consultation with the treating radiation oncologist. In one case (patient 2) we recommended the diet during RCT in an attempt to stop continuing weight loss. All patients were told, however, of the limited clinical experience with this diet in the context of cancer treatment and informed about potential side effects such as headaches, light-headedness, constipation/diarrhea and weight loss. Exclusion criteria were defined a priori as Karnofsky index less than 80, type I diabetes, pregnancy, pace maker, cognitive impairments or rare metabolic diseases that would contradict a KD such as defects in key enzymes of ketogenesis, ketolysis, gluconeogenesis or fatty acid oxidation. At study entry, all patients gave written informed consent to collect and publish their data. In particular, all patients agreed that their data could be used for scientific purposes such as publication in a scientific journal. Besides the KD and QoL assessment none of the interventions were unusual for a patient undergoing RCT in our clinic. All these procedures were performed according to the ethical standards of the declaration of Helsinki and approved by an institutional review board.

### Body composition

Body weight and composition were assessed at study entry and in regular intervals thereafter (the aim was once per week) through bioimpedance analysis (BIA) on a seca 515/514 medical body composition analyzer (mBCA; seca Deutschland, Hamburg, Germany). The mBCA device has a standing platform with an integrated scale and a handrail system, with one pair of electrodes for each hand and foot (eight-electrode BIA). A current of 100 μA and a total of 19 frequencies between 1 and 1000 kHz are used to obtain impedance measurements. Instrumental precision is specified as 100 g for BW between 50 and 200 kg and 5 Ω for impedance of the left and right half of the body. Fat free mass (FFM), extracellular water (ECW) and total body water (TBW) are derived from predictive equations that have been validated against dual-energy X-ray absorptiometry, air-displacement plethysmography and D_2_O and NaBr dilution techniques, yielding root mean square errors of 1.9 kg for FFM, 0.8 l for ECW and 1.3 kg for TBW [12].

In order to minimize systematic errors, patients were always measured fasted and without having drunk for at least 10 h. Patients were also asked to void their bladder prior to each measurement. Following the manufacturer’s instructions, body height was measured to within the closest 5 mm with a seca 231 stadiometer before the patient stepped onto the mBCA platform and placed feet and hands on the corresponding electrodes. Besides body weight (BW), the mean electrical resistance (R) and reactance (X_C_) between left and right half of the body, and the quantities FFM, ECW and TBW derived therewith, the following parameters were recorded for each patient: fat mass (FM = BW − FFM), intracellular water (ICW = TBW − ECW), hydration (= ECW/ICW) and phase angle at 50 kHz (PA = arctan (X_C_/R)). The PA is of special interest for oncological and other critically ill patients, as small PA values have been shown to predict a poor prognosis [13, 14].

### Blood parameters

In all patients except patient 5, blood draws were performed at study entry, once during RT and in the week of treatment termination. Parameters of interest included a complete blood count as well as HDL and LDL cholesterol, BHB, insulin, insulin-like growth factor 1 (IGF1) and thyroid stimulating hormone (TSH). In patient 5 blood test results were requested from the hospital where the patient underwent chemotherapy. Starting with the fourth patient, we also used occasional finger-pricktests with a FreeStyle Precision device (Abbott Diabetes Care Ltd., Range Road, Witney, UK) in order to determine BHB and blood glucose concentrations.

### Ketogenic diets

Due to the limitations imposed by the ambulatory setting in our clinic, all KDs were self-administered with counselling at least once per week. Patients received short handouts providing basic information on KDs such as main foods to eat and avoid as well as sample menus. For more detailed information we also lent and recommended to buy a popular book on KDs for cancer patients [15]. In general we recommended to restrict CHO intake to <50 g/day and emphasized the intake of olive oil, coconut oil, butter, ghee, and fatty fish, cheese, meat and non-starchy vegetables. To monitor ketosis, patients were provided with urine strips (Ketostix^®^, Bayer Consumer Care AG, Basel, Switzerland) and a diary for protocolling their measurements, preferably done in the afternoon. Patients were also asked to record the quantity of all foods eaten for at least 2 days in order to determine their actual energy and macronutrient intake.

### Questionnaires

QoL was assessed using the EORTC QLC-C30 questionnaire version 3.0 together with its disease-specific modules [16]. At termination of RT, each patient received a short non-validated questionnaire addressing several aspects of their subjective feeling with regard to the KD.

### Statistical analysis

For sake of simplicity we assume a linear relationship between body composition changes and time that we model using Bayesian linear regression [17]. If we have *N* measurements $y_{i} \;\left( {i = 1, \ldots ,N} \right)$ made at time points *t*_*i*_ then we assume

$$y_{i} \sim N\left( {\mu_{i} ,\tau^{ - 1} } \right), \quad \mu_{i} = \alpha + \beta t_{i} ,$$

where α and β are the intercept and slope of the linear relation, modelled with a flat prior distribution, and τ is the inverse of the residual variance σ^2^. Since we do not know σ exactly, we model it as a random variable drawn from a uniform prior distribution in the range [0.01, 10] times the unit of the parameter under consideration. Changes in the parameters of interest were considered significant if the 95 % highest posterior density interval for the slope β excluded 0. In the figures two-sided p values are also reported in order to put the results into a frequentist framework. Statistical analysis was performed using R version 3.0.2 and OpenBUGS version 3.2.2.

## Results

An overview of patient and treatment characteristics is given in Table 1. Analysis of the food diaries indicated that all patients consumed <50 g CHO per day as recommended (Table 2). Average energy intake from fat was 73 % (SD 5 %) which was below the suggested 80 %, and the ketogenic ratio was less than 2:1 in all patients. We note, however, that usage of cooking fats or salad dressings was not reported except by patient 5, so that real fat intake was probably higher.

**Table 1:** Baseline patient and treatment characteristics

	Patient 1	Patient 2	Patient 3	Patient 4	Patient 5	Patient 6
Sex	F	M	M	M	M	F
Tumor entity	Breast (adenocarcinoma)	Prostate (adenocarcinoma)	Rectum (adenocarcinoma)	Rectum (adenocarcinoma)	Primary: lung (small cell neuroendocrine); secondary: liver + bone (left shoulder)	Rectum (adenocarcinoma)
Tumor stage	IA	IV	IIA	IIIB	IV	IIIA
Age (years)	50	74	54	74	70	40
Height (m)	1.77	1.70	1.82	1.72	1.76	1.65
Baseline weight (kg)	88.9	67.5	76.4	89.4	114.0	55.9
Main reasons for KD	Anticipated fat mass reduction; anti-cancer effects	Anticipated body weight preservation	Anticipated anti-cancer effects	Anticipated anti-cancer effects; patient refusal of surgery	Anticipated anti-cancer effects; possibility to take action by oneself	Anticipated anti-cancer effects

The overall tumor staging refers to the AJCC 7th edition

**Table 2:** Details of the KDs based on food diaries

	Patient 1	Patient 2	Patient 3	Patient 4	Patient 5	Patient 6
Time on KD (days)	36	32	42	55+	73	51
Average CHO intake (g/day)	29	44	47	14	35	25
Average protein intake (g/day)	105	97	135	75	106	83
Average fat intake (g/day)	166	168	154	107	248	157
Average energy intake (kcal day^−1^)	2030	2076	2114	1402	2796	1843
Energy from fat (%)	74	73	66	69	80	76
Ketogenic ratio	1.2:1	1.2:1	0.8:1	1.2:1	1.8:1	1.4:1

The ketogenic ratio gives the weight of fat relative to the combined weight of CHO and protein in the diet. For convenience, we used a caloric equivalent of 4 kcal for 1 g of protein. Note, however, that the caloric content of protein depends on its amino acid composition, with high-quality protein providing less energy since more of its amino acids are used for body protein syntheses instead of energy production

QoL, a few selected symptoms and diet-related outcomes concerning wellbeing and practicability of the diet are summarized in Table 3. Four of the six patients were skeptical about consuming mainly fat. There was consensus between all patients that the KD was more satiating than their previous diet, and two patients reported an additional appetite loss during the last week of RT. However, the general subjective feeling on the diet was rated as “good”, and all patients decided to continue with a low CHO or ketogenic diet after RT. Global health status and total functional score remained fairly unchanged, while some symptom scores increased, eventually more due to RT-induced early side effects than by the diet itself. All patients completed RT without treatment breaks as normal. There were no side-effects from fasting and not drinking prior to each BIA measurement.

**Table 3:** Quality of life, subjective wellbeing and practicability of the KD

	Patient 1	Patient 2	Patient 3	Patient 4	Patient 5	Patient 6
How do you rate the practicability of the diet?	Easy	Difficult (at the beginning)	Difficult	Easy	Difficult	Easy
Did you experience changes in your appetite?	Less hunger	Less hunger	Less hunger	Less hunger	Less hunger	Less hunger
Did you have concerns eating mainly fat?	Yes	Yes	Yes	No	No	Yes
Have you been able to perform your usual level of exercise? If not was it due to the diet or RT?	Less training due to RT; less condition and strength attributed to RT	No problems (the only exercise was hiking)	Minor problems (not during exercise, but more exhausted afterwards) attributed to diet	No exercise	No exercise	No problems
What was your general feeling during the diet (besides exercise)?	Good	Good	Good; not good during the last 10 days of RT	Good	Good	Very good
What has improved most?	Vanishing of chronic migraine headaches; no more postprandial tiredness	Nothing special	Nothing special	Nothing special	Less snoring; feeling of taking self-responsibility	Good mood, euphoria; engagement with the diet distracted from the actual disease
How will your diet look like after RT?	CHO restricted but not so strict	CHO restricted but not so strict	CHO restricted but not so strict	Ketogenic diet	Ketogenic diet	CHO restricted but not so strict
Global health status/ QoL						
Baseline	91.7	83.3	66.7	66.7	NA	83.3
End of RT	75.0	83.3	58.3	66.7	NA	75
Total functional score						
Baseline	593.3	540.0	633.3	604.4	NA	711.1
End of RT	683.3	560.0	575.0	751.7	NA	725
Fatigue						
Baseline	55.6	22.2	44.4	11.1	NA	11.1
End of RT	33.3	22.2	44.4	22.2	NA	33.3
Nausea and vomiting						
Baseline	0	0	0	0	NA	0
End of RT	16.7	0	16.7	0	NA	0
Appetite loss						
Baseline	0	0	0	0	NA	0
End of RT	0	66.7	66.7	0	NA	33.3
Constipation						
Baseline	0	0	0	0	NA	0
End of RT	0	0	0	0	NA	0
Diarrhea						
Baseline	0	100	0	0	NA	66.7
End of RT	0	100	66.7	0	NA	66.7

Outcomes of EORTC QLQ-C30 are summarized by global health status, total functional score and symptom scales including scores from site-specific questionnaires. The questions in the left column stem from our own non-validated questionnaire in which answers had to be chosen from a predefined list of items

**Fig. 1:**
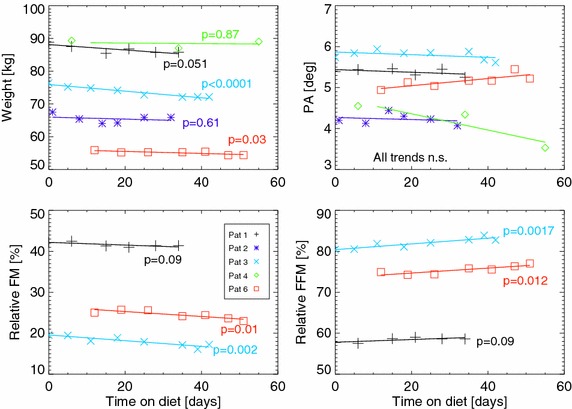
Body composition changes over the course of combined RT and KD. The *symbols* represent measured data in relation to the time on the KD, while the *lines* are drawn only for the duration of RT based on the linear regression fits with p values indicated for each fitted trend. Estimation of FM and ICW was unreliable for patients 2 and 4, so their data was omitted from the *lower panels*

**Fig. 2:**
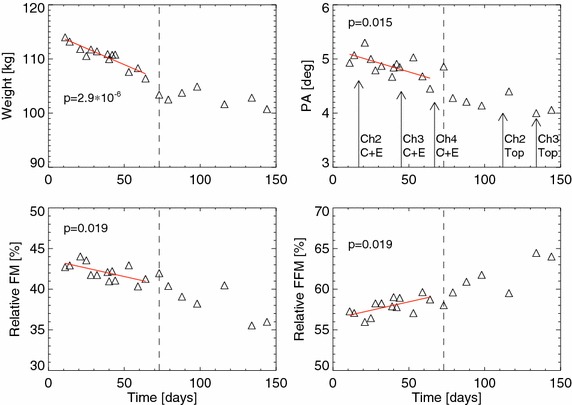
Similar to Fig. 1, but only for patient 5. Each *panel* includes a *vertical line* which indicates the transition from a KD to a low-CHO diet. Trends have only been computed and drawn for the time on the KD. The *arrows* indicate the start of 3 day chemotherapy cycles (Ch) with cisplatin/etoposide (C + E) or topotecan (Top), respectively. RT for a metastasis in the right humerus occurred between C + E cycles 2 and 3

The results of the body composition analysis are given in Table 4 and displayed in Figs. 1 and 2. Changes in patient 5 were only computed for the 73 days that this patient followed a KD (see below for more details). Weight loss occurred in all patients during the combined RT/KD, but changes were only significant in patients 3 and 5. Unfortunately, patients 2 and 4 had metallic parts within their bodies that made body composition estimates unreliable. BIA estimates in the remaining four patients suggested that body mass loss was mainly composed of FM which decreased significantly in patients 3, 5 and 6 in absolute as well as relative terms. In contrast, FFM did not change significantly in absolute terms but increased significantly relative to body weight in patients 3, 5 and 6 (Table 4). No significant changes in ECW, TBW or hydration status (ECW/ICW) where observed, but ICW decreased significantly in patients 1, 3 and 5 and increased significantly in patient 6. No significant changes in PA occurred with the exception of a decrease in patient 5 (Fig. 2).

**Table 4:** Estimated slopes and 95 % highest posterior density (HPD) intervals for linear regression on the body composition quantities against time on the KD

Slope β	Patient 1	Patient 2	Patient 3	Patient 4	Patient 5 ^a^	Patient 6
BW (kg/week)	−0.58 [−1.35, 0.18]	−0.21 [−1.59, 1.17]	−0.71 [−0.90, −0.53]	−0.06 [−2.29, 2.18]	−0.84 [−1.07,−0.61]	−0.22 [−0.43,0.01]
Relative BW (%/week)	−0.64 [−1.45,0.21]	−0.31 [−2.25,1.65]	−0.93 [−1.18,−0.68]	−0.07 [−2.33,2.25]	−0.74 [−0.94,−0.54]	−0.38 [−0.78,0.01]
PA (°/week)	−0.02 [−0.10,0.05]	−0.02 [−0.16,0.12]	−0.02 [−0.07, 0.03]	−0.14 [−1.8, 1.6]	−0.06 [−0.08,−0.04]	0.06 [−0.002,0.12]
FM (kg/week)	−0.40 [−1.02,0.22]	NA	−0.49 [−0.68,−0.30]	NA	−0.68 [−0.90,−0.45]	−0.28 [−0.48,−0.09]
Relative FM (%/week)	−0.21 [−0.63, 0.23]	NA	−0.49 [−0.75, −0.23]	NA	−0.29 [−0.54,−0.04]	−0.42 [−0.76, −0.10]
FFM (kg/week)	−0.13 [−0.61,0.35]	NA	−0.22 [−0.45,0.02]	NA	−0.17 [−0.53,0.21]	0.07 [−0.18,0.32]
Relative FFM (%/week)	0.23 [−0.14,0.61]	NA	0.49 [0.37,1.46]	NA	0.29 [0.04,0.54]	0.42 [0.09,0.75]
ECW (l/week)	−0.023 [−0.31, 0.26]	NA	−0.063 [−0.18, 0.054	NA	−0.004 [−0.18,0.18]	−0.069 [−0.28, 0.14]
ICW (l/week)	−0.15 [−0.30, −0.01]	NA	−0.15 [−0.27, −0.06]	NA	−0.13 [−0.25, −0.008]	0.18 [0.04, 0.32]
Hydration (ECW/ICW) (%/week)	0.46 [−0.49, 1.42]	NA	0.16 [−0.22, 0.53]	NA	0.39 [−0.15,0.91]	−1.23 [−2.65, 0.19]

Trends are considered significant if the 95 % HPD interval excludes 0, with positive slopes indicating an increase, negative slopes a decrease in the quantity under consideration

^a^ Trends in patient 5 where only computed for the 73 days he followed a KD

The results of the blood tests are given in Table 5. No clear trends could be observed in the biochemical parameters, except for a rise of BHB concentrations after baseline blood testing, as expected. It is noticeable that some patients had low BHB and high glucose concentrations despite their food diaries and self-reports during consultation indicating strict adherence to a KD regime. When all 32 fasting measurements were pooled together (lab tests and finger-prick tests) there was an indication for a significant negative correlation between BHB and glucose concentration (Spearman’s ρ = −0.35, p = 0.05).

**Table 5:** Biochemical blood parameters

Patient	Patient 1			Patient 2			Patient 3			Patient 4			Patient 5				Patient 6		
Time on KD (days)	0	21	34	0	21	32	0	18	42	6	34	54	0	17	45	66	12	33	51
HbA1c (%)	5.7 (+)	5.5	5.4	NA	6.0 (+)	5.8 (+)	NA	5.1	5.3	5.3	5.5	5.5	5.7	5.5	NA	5.2	5.4	5.6	5.3
Glucose (mg/dl)	85	95	109	127 (+)	124 (+)	134 (+)	92	91	98	91	103	104	96	88	86	94	84	92	92
BHB (mmol/l)	0.03	0.33 (+)	0.24	0.08	0.20	0.11	0.22	1.17 (+)	0.43 (+)	0.81 (+)	1.31 (+)	0.98 (+)	NA	NA	NA	NA	0.53 (+)	0.41 (+)	0.68 (+)
LDL-C (mg/dl)	NA	126	137	53	60	83	112	92	62	84	68	87	63	78	98	84	177	174	189
HDL-C (mg/dl)	NA	57	59	45	47	64	78	83	85	56	59	65	51	45	57	33 (−)	73	93	89
Triglycerides (mg/dl)	NA	66	80	87	83	44	50	69	54	72	51	51	92	217 (+)	103	134	48	35	47
AST (U/l)	16	23	20	28	27	46	19	15	16	32	20	22	47	54 (+)	44	40	22	21	18
ALT (U/l)	11	13	12	51 (+)	42 (+)	50 (+)	18	21	17	28	NA	22	90 (+)	130 (+)	93 (+)	91 (+)	20	30	18
Gamma-GT (U/l)	10	8	9	367 (+)	275 (+)	428 (+)	25	20	14	33	NA	21	97 (+)	93 (+)	131 (+)	155.7 (+)	11	9	10
Creatinine (mg/dl)	0.74	0.70	0.72	0.63 (−)	0.75	0.77	0.89	0.90	0.92	1.01	1.00	0.78	1.02	1.02	1.14	1.07	0.69	0.68	0.72
CRP (mg/l)	1.4	NA	1.6	0.8	1.2	0.8	1.1	4.2	3.8	4.4	21.2 (+)	10.5 (+)	0.4	0.64	0.64	3.51	6.9 (+)	0.7	5.9 (+)
Insulin (mU/l)	12.6	8.7	15.6	12.0	16.7	11.1	5.3	6.1	6.7	11.0	15.5	13.1	NA	NA	NA	NA	4.2	3.6	3.6
IGF-1 (ng/ml)	202	187	203	272 (+)	266 (+)	228	101	99	131	144	177	132	NA	NA	NA	NA	115	184	164
TSH (mU/l)	NA	NA	NA	2.51	2.03	2.54	0.98	0.79	0.90	0.68	0.63	0.72	1.8	1.5	NA	1.8	3.89	2.64	2.77
Free T3 (pg/ml)	NA	NA	NA	2.89	2.16 (−)	2.53 (−)	3.58	2.71	3.01	2.65	2.63	2.54 (−)	NA	NA	NA	NA	2.44 (−)	2.07 (−)	2.20 (−)
Free T4 (ng/dl)	NA	NA	NA	1.18	0.99	1.07	1.23	1.22	1.15	1.70	1.56	1.39	NA	NA	NA	NA	1.06	0.88 (−)	0.98

Values outside the laboratory reference rages are indicated as either too low (−) or too high (+)

Below, a brief description of interesting results for some individual patients is given.

### Patient 2

Patient 2 had multiple comorbidities and started adjuvant RT with simultaneous hormone therapy on September 18th 2014. After 11 fractions the patient started a KD supplemented with 10 g/day of MAP amino acids (dr. reinwald gmbh + co kg, Schwarzenbruck, Germany) in the hope of improving his physical condition and preserving FFM since he had lost 5 kg during the 4 months since surgery and suffered from diarrhea. The patient had difficulties with food selection mainly because he had been told to avoid fatty foods for several years and the KD presented a totally new way of eating. Due to these difficulties and a further weight loss of 3.5 kg after 2 weeks we prescribed him a ketogenic liquid drink (Keto-Drink, Tavarlin, Darmstadt, Germany) providing 4.0 g of CHO, 18.5 g of protein and 42.0 g of fat (500 kcal) per 250 ml serving. His weight subsequently increased but remained approximately 1.5 kg below baseline at the end of RT. Although the patient reported some positive urine test results, blood tests revealed no elevated serum BHB levels and blood glucose levels remained elevated above 120 mg/dl. At 4 months follow-up, the patient had regained 6 kg of BW and felt subjectively very good. His PA was 4.26° which was not different from the mean value of 4.24° during the KD phase.

### Patient 3

This patient started the KD concurrently with neoadjuvant RCT on 9th October 2014 and remained on it until the end of RCT. He had 35 of 39 positive test results for acetoacetate in the urine. Four weeks into RCT he developed radiation enteritis and grade 1/2 diarrhea with abdominal pain which was treated with Imodium and partly switched to obstipation thereafter. Concurrently he developed an aversion against fatty foods which made dietary compliance difficult. The symptoms resolved soon after termination of RCT. Seven weeks after RCT the patient underwent surgery where the tumor had regressed as expected (Grade 2 on the Dvorak grading scale). Final tumor stage was ypT2 ypN0 (0/13) L0 V0 Pn0 GX R0. After surgery the patient received dietary advice from the department of internal medicine and stopped eating low carb. At 4 month follow-up the patient’s weight was basically unchanged compared to the end of RT (BW 72.3 kg), but he had gained approximately 1.5 kg of FM and lost 1 kg of FFM.

### Patient 4

This patient started the KD 11 days prior to the start of RCT, mainly because he refused surgery and hoped that the diet would help in fighting his cancer. He reported positive testing for urinary acetoacetate on all 39 days of treatment. All laboratory and finger-prick blood tests confirmed that he was in ketosis with BHB concentrations exceeding 0.7 mmol/l. Blood sampling in week 4 of RCT revealed a high CRP value (Table 5); upon request the patient reported an acute infection. An MRI scan after 24 fractions showed no more signs of rectal wall thickening or lymph node enlargements, but a conspicuous zone in the dorsal prostate. One day before his last RT fraction BIA indicated a PA of only 3.6°. A new measurement was performed on the following day, confirming the result (data point shown in Fig. 1). Final blood testing yielded elevated PSA and CRP concentrations of 13.04 ng/ml and 10.5 mg/l, respectively, hinting towards an inflammatory process. Tolerability of RCT was excellent with perianal radiodermitis at most grade 1 and no gastrointestinal problems. At 4 months follow-up the patient was still following the KD. There were no late toxicities, no hints towards a residual tumor or pathologic lymph nodes from an MRI exam, and PSA had declined to 5.63 ng/ml.

### Patient 5

This patient sought our dietary advice after having been diagnosed with metastatic small cell lung cancer and having received his first cycle of cisplatin/etoposide chemotherapy. He started the diet 7 days after his first chemotherapy cycle. Laboratory testing performed at the start of each chemo cycle showed the disappearance of glucosuria that had been present at the first cycle and development of ketonuria while on the diet; a drop of HbA1c and elevated BHB levels in a total of 14 fasted and postprandial finger prick tests (mean 0.68, SD 0.58 mmol/l) indicated compliance with the diet. He had to interrupt the diet for the 3 days of his second chemo cycle because the hospital was not able to provide him a KD. At day 24 on the diet he began palliative RT of a metastasis in the right humerus that ended 1 day before his third chemo cycle. On two occasions during RT we tested the impact of two different liquid ketogenic drinks on BHB levels: (1) 250 ml of Keto-Drink, providing 42.0 g of fat of which 8.8 g are medium chain triglycerides or (2) 250 ml of betaquick^®^ (Vitaflo Pharma GmbH, Bad Homburg, Germany), providing 0 g CHO, 0 g protein and 52.5 g fat of which 50 g are medium chain triglycerides (473 kcal). BHB was measured in the fasted state and 1 h postprandial with finger-prick tests. In condition (1), BHB concentrations rose from 0.3  to 0.9 mmol/l and in condition (2) from 0.4 to 1.1 mmol/l, showing that both drinks were able to significantly enhance ketosis.

This patient was followed with regular BIA and finger-prick measurements beyond his RT treatment period in our clinic. A restaging CT/MRT was performed on day 66 of his diet. This showed a slight progression of his primary tumor, slight regression of pulmonary lymph nodes, multiple slightly progressive as well as newly formed liver metastases and a newly formed osseous lesion in T12. One day later he started the fourth cycle of cisplatin/etoposide. At day 73 on the diet he switched to a less restricted low carbohydrate diet with main focus on avoidance of sugar. A DOTATOC-PET performed 3 weeks later indicated at most a slight uptake of the tracer, so that radionuclide therapy was no further option. It was decided to change his chemotherapy to 1.5 mg/m^2^ topotecan. Interestingly, an in vitro chemo-sensitivity test in which circulating epithelial tumor cells where extracted by the MAINTRAC method and exposed to eutherapeutic concentrations of chemotherapeutic drugs [18] was requested by a treating naturopath and showed that tumor cell viability reduction after treatment with carboplatin was less than 10 %, while topotecan achieved a 90 % reduction. Nevertheless, the patients experienced rapid disease progression during six further cycles of topotecan and died 11 months and 5 days after his diagnosis.

## Discussion

In this paper we describe our initial experience with the first six patients who undertook a KD during RT. While the small sample size, short duration of follow-up and lack of a control group are obvious limitations for the interpretation of our results the strength of our data is that patients were followed prospectively and many measurements of clinical relevance were made. A main result is that the KD intervention within the context of a busy community hospital setting was feasible, but posed some challenges, the main one being patient compliance which is hard to control within an ambulatory setting. From our experience with the six cases presented here we have learned that three measures would help to maximize compliance and high ketosis: (1) frequent individual counselling by a dietician experienced with KDs; (2) provision of ketogenic formulas and meals; (3) offering cooking classes. These three steps have now been implemented in our clinic for the care of future patients who wish to undertake a KD during RT.

Importantly, no diet-related side effects occurred in our patients who finished RT without breaks and rated their subjective feeling on the diet as “good”. Furthermore no negative effects on blood parameters were detectable, contradicting the account of some authors who describe the KD as unhealthy or even dangerous. It is likely that limiting the duration of a KD to a short timeframe such as the period of RT limits any negative impact on blood parameters. Weight loss could be a concern, however, although in our study this has to be interpreted within the context of RCT that by itself can lead to BW loss of a similar magnitude as observed in our patients [19]. While theoretically a KD has both appetite stimulating and appetite suppressing mechanisms, the interaction of these mechanisms as well as the common experience in humans speaks more for an overall anorexigenic effect [20], consistent with the reports of our patients.

Our BIA data indicate that weight loss was mainly due to FM loss during the diet phase. PA was also basically unaltered with the exceptions of patient 5 who had extensive disease with a rapidly growing tumor and the steep decrease of PA at the final measurement in patient 4 that remains open for interpretation. PA is an important measure of overall health, muscularity and nutritional status on the cellular level [21] and it is not uncommon for PA to decrease during RT [19].

While a decrease in FM can even be rated as beneficial for some patients [22] there is strong evidence that skeletal muscle mass is the crucial component for long-term prognosis and QoL [23]. It would be ideal for a diet of cancer patients to both counteract protein catabolism and stimulate protein anabolism. The KD might be such a “cancer-specific diet” [10]. Regarding catabolism, studies have shown that ketosis decreases urinary nitrogen losses and muscle mass breakdown in both healthy individuals [24] and undernourished cancer patients [25]. Regarding anabolism it should be considered that different protein sources differ in their net nitrogen utilization (NNU), i.e., the percentage of amino acids that—after digestion—get utilized in the anabolic pathway to build new body protein without energy production or nitrogen loss [26]. Proteins with lower NNU provide a larger percentage of amino acids that get deaminated in the catabolic pathway, delivering more precursors for endogenous glucose production via gluconeogenesis as well as nitrogen catabolites that have to be detoxified by the liver. For this reason we advised our patients to consume high quality proteins such as fish and eggs and began substituting them with MAP which has been shown to have a NNU of 99 % when used as the sole protein source in healthy individuals [26].

Focussing on high quality protein makes particular sense in the context of a KD where the ketosis-repressing properties of dietary protein have long been known. The classic KD used for treating intractable epilepsy in children has therefore been based on calorie restriction and a ketogenic ratio of at least 3:1 in order to maximize ketosis and its therapeutic efficacy [27], with the downside of severe protein restriction. The ketogenic ratio in our patients ranged from approximately 1:1–2:1 (but probably higher due to not reporting of cooking fats and dressings) which allowed for ample protein intake and good tolerability, but might have been too low to considerably elevate BHB concentrations in some patients. Our experiments with patient 5 suggest that ketogenic drinks, especially enriched with medium chain triglycerides, provide an easy way to transiently increase the ketone to blood glucose ratio.

Given the large variety of human tumors and the fact that most cancer patients are old and/or present with weight loss, sarcopenia, diabetes and other metabolic comorbidities (including those induced by their tumor) it is presently unclear what the ideal ketogenic ratio for an individual cancer patient is. Thomas Seyfried’s group has emphasized the importance of maximizing the ketone to blood glucose ratio [28]. Can we achieve a good compromise between maximally lowering this ratio and providing sufficiently high protein and energy intake in order to maintain important muscle tissue? Can we also implement additional calorie restriction in certain patients to enhance efficacy without a negative impact on body composition as indicated by one case report [29]? These questions must be addressed with human data. For our part, the initial observations reported here motivated us to initiate a randomized study on the feasibility and effects of a KD intervention on body composition, blood parameters and QoL. The protocol of this study called KETOCOMP (ClinicalTrials.gov identifier: NCT02516501) is provided in a companion article [30].

## Conclusions

We presented a variety of prospectively collected measurements on the first six consecutive patients that undertook a KD during RT treatment in our clinic within a busy community hospital. No serious diet-related side effects occurred, and subjective feeling on the diet was rated as good. Ketosis was not as pronounced as it is usually reported for protein- or calorically restricted KDs, and blood sugar levels did not decrease significantly. This might indicate problems with either compliance or prescribed diet composition which is discussed within the context of the ideal diet composition for cancer patients aiming at the preservation of muscle mass. Importantly, although significant weight loss occurred in some patients, our BIA data indicate that weight loss was mainly due to fat mass loss. From these observations we implemented additional measures of diet counselling and initiated a randomized study (ClinicalTrials.gov identifier: NCT02516501) the protocol of which is published in a companion paper [30].

## Abbreviations

BHB: beta-hydroxybutyrate
BIA: bioimpedance analysis
CHO: carbohydrate
ECW: extracellular water
FFM: fat free mass
FM: fat mass
ICW: intracellular water
KD: ketogenic diet
NNU: net nitrogen utilization
PA: phase angle at 50 kHz
QoL: quality of life
RCT: radiochemotherapy
RT: radiotherapy
TBW: total body water
